# REGγ deficiency suppresses tumor progression via stabilizing CK1ε in renal cell carcinoma

**DOI:** 10.1038/s41419-018-0646-2

**Published:** 2018-05-24

**Authors:** Shaojun Chen, Qingwei Wang, Longsheng Wang, Hui Chen, Xiao Gao, Dongkui Gong, Junjie Ma, Syeda Kubra, Xudong Yao, Xiaotao Li, Lei Li, Wei Zhai, Junhua Zheng

**Affiliations:** 1 Department of Urology, Shanghai Tenth People’s Hospital, Tongji University, 200072 Shanghai, China; 2 Shanghai Key Laboratory of Regulatory Biology, Institute of Biomedical Sciences, School of Life Sciences, East China Normal University, 200241 Shanghai, China; 3 Department of Molecular Molecular and Cellular Biology, Dan L. Duncan Cancer Center, Baylor College of Medicine, Houston, Tx 77030 USA; 4 Department of Urology, Renji Hospital, School of Medicine, Shanghai Jiao Tong University, 200127 Shanghai, China; 5 Department of Urology, Shanghai First People’s Hospital, School of Medicine, Shanghai Jiao Tong University, 200080 Shanghai, China

## Abstract

Renal cell carcinoma (RCC) is the most common malignant disease of kidney in adults. The proteasome activator REGγ was previously reported to promote the degradation of multiple important regulatory proteins and involved in the progression and development of numerous human cancers. Here, we first reported that REGγ was upregulated in RCC and its upregulation was correlated with a poor prognosis in RCC patients. REGγ depletion obviously suppressed RCC cells proliferation in vitro and in vivo. Notably, casein kinase 1ε (CK1ε) was identified as a novel target of REGγ and knockdown of CK1ε effectively abolished the effect of REGγ depletion on RCC cells growth. Importantly, we also observed that REGγ depletion activated Hippo signaling pathway via stabilizing CK1ε in RCC, indicating the cross-talk between REGγ/CK1ε axis and Hippo pathway during RCC development. In conclusion, our findings suggested that REGγ played a pivotal role in the development of RCC and maybe helpful to identify new therapeutic strategies in the treatment of RCC.

## Introduction

Renal cell carcinoma (RCC) is one of the most common malignant diseases of kidney and the incidence of RCC is steadily increasing by 2–4% each year in recent years^1^. In 2016, about 62,700 new cases and 14,240 deaths were estimated to occur in the United States^2^. Surgical resection is generally used as the standard approach to remove localized RCC and the 5-year overall survival rate of non-metastatic RCC is approximately 55%^3^. However, nearly 20-40% of post-surgery patients still develop local recurrence or distant metastasis and the 5-year overall survival rate of metastatic RCC is only 9%^4^. What is worse, approximately 20-25% of first diagnosed RCC patients have already reached the metastatic phase^5^. Therefore, it is crucial to explore novel molecules involved in the progression of RCC, so as to identify new therapeutic targets for RCC treatment.

REGγ, also known as PSME3 or PA28γ, is a member of the 11S proteasome activator family that regulates the degradation of many important regulatory proteins in an ubiquitin- and ATP-independent manner^6, 7^. Indeed, REGγ was reported to be involved in the regulation of various cellular processes. For example, REGγ-deficient mice displayed a significantly reduce in body size and REGγ-deficient mouse embryonic fibroblasts (MEFs) have impeded entry from G to S phase in the cell cycle^8, 9^, indicating its regulation in cell proliferation and cell cycle transition. Accumulating evidence indicated that REGγ was overexpressed in multiple human cancers including breast cancer, thyroid cancer and lung cancer^10–12^. However, the expression pattern and role of REGγ in RCC remains elusive.

The casein kinase 1 (CK1) family, which exists in seven isoforms (α, β, γ1, γ2, γ3, δ, and ε) in mammals, is one of the serine/threonine protein kinase families^13, 14^. CK1 kinases participate in multiple cellular processes such as cell division, differentiation, and apoptosis^13, 15^. In recent years, an increasing number of studies have disclosed the role of CK1ε in cancer. Existing reports showed that patients with higher CK1ε expression had a considerably better outcome than patients with lower CK1ε expression in oral squamous cell carcinoma^16^, breast cancer^17^, and colorectal cancer^18^. Additionally, Fuja et al. reported that CK1ε expression was reduced in poorly differentiated tumors and increased in more benign ductal cell carcinoma in situ^19^. These indicated the important roles of CK1ε in cancer initiation and progression.

In this study, we investigated the role of REGγ and its potential mechanism in RCC for the first time. We found the expression level of REGγ was obviously increased in RCC and its high expression was correlated with a poor prognosis in RCC patients. In addition, knockdown of REGγ significantly inhibited proliferation, migration, and invasion and enhanced apoptosis in RCC cells. Furthermore, we demonstrated that knockdown of REGγ activated Hippo signaling pathway via stabilizing CK1ε in RCC. Our results collectively suggested that REGγ played an important role in the development of RCC and that REGγ may act as a novel therapeutic target in RCC treatment.

## Materials and methods

### Clinical tissue samples

This study was approved by the Ethics Committees of Shanghai Tenth People’s Hospital and written informed consent was obtained from each patient. A total of 81 RCC tissues and 30 corresponding normal kidney tissues were obtained from primary RCC patients who underwent radical nephrectomy at the department of urology, Shanghai Tenth People’s Hospital between 2008 and 2012. None of the patients received any preoperative treatment. The follow-up period was at least 60 months. All tissue specimens were snap-frozen immediately in liquid nitrogen and stored at −80 °C until use.

### Cell culture

Human RCC cell lines 786-O and caki-1 were cultured in RPMI-1640 medium (Gibco BRL, Grand Island, NY, USA). The normal renal tubular epithelial cell line (HK-2) was cultured in F-12 medium (Gibco). Other cell lines were all cultured in DMEM (Gibco). All media were supplemented with 10% fetal bovine serum (Gibco), 100 U/ml penicillin, and 100 mg/ml streptomycin (Gibco). Cells were maintained in a humidified incubator at 37 °C with 5% CO_2_. The four RCC cell lines (A498, 786-O, ACHN, caki-1) and HK-2 were obtained from Cell Bank of the Chinese Academy of Sciences (Shanghai, China). The REGγ-inducible 293 WT or N151Y cell lines were previously reported^20^. MEFs were isolated from E13.5-day REGγ+/+and REGγ−/− mouse embryos, and immortalized MEFs were described previously^20^. The stable REGγ knockdown RCC cell lines ACHN and A498 were generated by integration of retroviral shREGγ vectors specific for REGγ or a control gene (GFP) from OriGene (Rockville, MD), which was performed as previously described^21^. Transduced cells were selected in puromycin (Invitrogen, CA, USA).

### Immunohistochemistry

IHC analysis was carried out as previously described^22^. Briefly, tissue samples were fixed with 4% paraformaldehyde, dehydrated through a graded series of ethanol, and embedded in paraffin. The 4-μm sections were deparaffinized, rehydrated, and stained with hematoxylin and eosin (H&E). For IHC, the tissue sections were processed for antigen retrieval, blocked with goat serum and incubated with primary antibody at 4 °C overnight. Subsequently, the sections were incubated with goat anti-rabbit secondary antibody for 20 min at room temperature and then for 30 min with Streptavidin-HRP peroxidase. Diaminobenzidine (DAB)-H_2_O_2_ was used as a substrate for the peroxidase enzyme. Then, the sections were stained with hematoxylin and dehydration. The primary antibodies used for IHC analysis were purchased from Invitrogen (REGγ), Abcam (CK1ε and ki-67), Proteintech (YAP), and Cell Signaling Technology (p-YAP).

Immunoreactivity for REGγ in RCC tissues was independently evaluated by two investigators (SJC and LSW). A semiquantitative scoring criterion was used, in which the staining index (values 0–12) were calculated by multiplying the staining intensity and the positive cells proportion^23^. The intensity of positive staining was scored as no-staining 0; weak 1; moderate 2; strong 3. The proportion of immune-positive cells was scored as 0–5% 0, 6–25% 1, 26–50% 2, 51–75% 3, >75% 4. Finally, cases were classified into two different groups: low expression group (score 0–6), and high expression group (score 7–12).

### Western blot

Total protein was extracted from tissue samples or cultured cells by using cold RIPA buffer (Beyotime Biotechnology, Shanghai, China) with protease inhibitor cocktail (Sigma-Aldrich, St. Louis, MO, USA) on ice. Protein concentration was measured by bicinchoninic acid (BCA) protein assay kit (Pierce, Rockford, IL, USA). Equal amount of protein was separated by 9–11% SDS-PAGE and transferred onto nitrocellulose membranes (Millipore, MA, USA). Membranes were blocked with 5% fat free dry milk in PBS for 1 h at room temperature and incubated with primary antibody at 4 °C overnight. After washing with PBS-T, then membranes were incubated with a fluorescent-labeled secondary antibody (Jackson Immuno Research) for 1 h at 4 °C. Finally, the specific signals were visualized by a LI-COR Odyssey Infrared Imaging System. The intensity was determined using Image J software. The primary antibodies used for western blot (WB) were purchased from Invitrogen (REGγ), BD Biosciences (CK1ε), sigma (HA, Flag), Abcam (β-actin, GAPDH), Proteintech (MST1, YAP), and Cell Signaling Technology (LATS1, p-LATS1, p-YAP).

### MTT assay

Cell viability was evaluated by the 3-(4, 5-dimethylthiazol-2-yl)-2, 5-diphenyl tetrazolium bromide (MTT) assay according to the manufacturer’s instruction. The cells were seeded into 96-well plates and incubated at 37 °C for different time periods (24 h, 48 h, 72 h, and 96 h). At each time point, 100 μl of full medium containing 0.5 mg/mL MTT (Sigma-Aldrich, St. Louis, Mo, USA) were added to each well and incubated for a further 4 h. Then, the media were discarded and 150 µl DMSO (Sigma) was added to resolve the crystals. The optical density (OD) values were measured at 490 nm (SpectraMax 190; Molecular Devices Sunnyvale, CA, USA)

### Colony formation assay

For colony formation assays, the cells were plated into 6-well plates at a density of 1 × 10^3^/well and cultured for 10 days. After washing twice with cold PBS, the cells were fixed with methanol and stained with 0.1% crystal violet (0.1% in 20% methanol). Images of stained tumor cell colonies were recorded with a digital camera.

### EdU incorporation assay

Cell proliferation was measured by the incorporation of 5-ethynyl-2’-deoxyuridine (EdU) during DNA synthesis using the Cell-Light™ EdU Apollo®488 In Vitro Imaging Kit(100 T) (Ribobio, Guangzhou, China) according the manufacture’s instruction. Briefly, the cells were seeded in 96-well plates the day before and incubated with 50 μM EdU for 2 h. Then, the cells were fixed with 4% paraformaldehyde and the cell nuclei were stained with Hoechst. Subsequently, the EdU positive cells were captured and quantified by fluorescence microscopy.

### Flow cytometry

Cell apoptosis rate was analyzed by flow cytometry with FITC-Annexin V Apoptosis Detection Kit (BD Biosciences, San Jose, CA) according to the manufacturer’s instruction. In brief, the cultured cells were collected, washed twice with cold PBS and resuspended in 1 × binding buffer. Then, cells were stained with 5 μl Annexin V-FITC and 5 μl propidium iodide (PI) in the dark for 15 min at room temperature. The apoptosis rate was measured by flow cytometry using BD FACS Calibur (Beckman Coulter, CA, USA).

For cell cycle analysis, the cultured cells were harvested, washed twice with pre-cooled PBS, and fixed in 70% ethanol at 4 °C overnight. Following that, the cells were washed and resuspended in PBS containing PI and 50 μg/ml RNase A (Sigma-Aldrich) in the dark at 37 °C for 30 min. Subsequently, the cell cycle analysis was performed by flow cytometry using BD FACS Calibur. Experiments were independently repeated three times.

### Wound-healing assay

Cells were seeded in six well plates and cultured to reach confluence. After starved overnight in medium with 1% FBS, the confluent monolayers were scraped with a 200 μL pipette tip to create a linear wound. Plates were washed with PBS to remove cell debris and then cultured with full medium for 48 h. Photographs of the wounds were taken with a phase contrast microscope (Olympus, Japan) and the horizontal distance between the sides of the wound was measured. Each experiment was performed three times independently.

### Transwell invasion assay

Matrigel-coated invasion chambers (BD Biosciences, USA) were used for transwell invasion assay according to the manufacturer’s protocol. Briefly, the cells were suspended in 200 μl serum-free medium and seeded on the upper chamber. The lower chamber was filled with 600 µL complete medium. After incubated for 48 h, the cells on the upper side of the membrane were wiped off with a cotton swab while cells on the lower side of the membrane were fixed with methanol and stained with crystal violet. The cells were photographed and counted in five random fields under the microscopic. Values were expressed as relative invasion ratio. This assay was performed in triplicate.

### RNA interference

Transient transfection was performed by using Lipofectamine 2000 (Invitrogen) following the manufacturer’s instructions. The sequences of si-REGγ were 5′-CAGAAGACUUGGUGGCAAATT-3′ (sense) and 5′-UUUGCCACCAAGUCUUCUGTT-3′ (antisense). The sequences of si- CK1ε were 5′-GCCAGAAGUAUGAACGGAUTT-3′ (sense) and 5′-AUCCGUUCAUACUUCUGGCTT-3′ (antisense). The sequences of si-NC were 5′-UUCUCCGAACGUGUCACGUTT-3′ (sense) and 5′-ACGUGACACGUUCGGAGAATT-3′ (antisense). siRNAs and the negative controls were all synthesized by GenePharma Co. Ltd (Shanghai, China).

### Immunoprecipitation

Early studies indicated that REGγ, as a proteasome activator, modulated multiple pathways by promoting the degradation of target proteins in various human cancer developments^24^. In our previous study, we reported that REGγ effectively interacted with CK1δ and drove its degradation in the regulation of aging^21^. To the best of our knowledge, among the seven members of CK1 family, CK1δ and CK1ε have the highest similarity with 98% identical in their kinase domain and 53% identical in their C-terminal regulatory domain^15, 25^. Therefore, we tested the hypothesis that whether REGγ facilitated the degradation of CK1ε. The pcDNA5-flag-REGγ plasmid was constructed previously^21^. The pCMV3-HA-CK1ε plasmid was constructed by Hanyin Co. (Shanghai, China). Plasmids were transfected into 293T cells as explained in the figures and immunoprecipitation was performed as previously described^21^. Antibodies against HA and Flag were purchased from sigma.

### Immunofluorescence (IF)

Cells were seeded onto glass coverslips placed in 24-well plates and cultured overnight at 37 °C. Then, the cells were fixed with 4% formaldehyde for 15 min and permeabilized with 0.1% Triton X-100 in PBS for 15 min at room temperature. After washing three times with PBS, the cells were blocked with 1% bovine serum albumin for 30 min and incubated with primary antibody against YAP (proteintech, 1: 250) overnight at 4 °C. Subsequently, the cells were incubated with an Alexa Fluor® 550-conjugated secondary antibody (Jackson Immuno Research, 1:1000) for 30 min and stained with DAPI (Sigma-Aldrich) for 5 min in the dark at room temperature. Finally, the cells were observed and photographed under a fluorescence microscope (Olympus, Tokyo, Japan).

### Xenograft model

A xenograft animal model was established in 6-week-old male BALB/c nude mice in the animal experimental center of East China Normal University. Briefly, a total of 2 × 10^6^ REGγ stable knockdown ACHN cells (shREGγ) or control cells (shNC) were implanted into the dorsal flanking sites of nude mice. After four weeks of tumor implantation, the mice were sacrificed and the tumors were isolated and immunohistological examined. The mice were provided by the SLAC Laboratory Animal Center (Shanghai, China) and cared in accordance with the NIH Guide for the Care and Use of Laboratory Animals.

### Statistical analysis

Statistical analyses were performed using SPSS software (version 17.0, SPSS, Inc., Chicago, IL, USA) and GraphPad Prism software (Version 6.0, GraphPad Prism Software Inc., San Diego, CA). Data were expressed as mean ± standard deviation (SD). Differences between two groups were analyzed using Student’s *t*-test or Mann–Whitney *U*-test. The correlation between REGγ expression and the clinical characteristics of RCC samples were determined using Pearson’s Chi-square test. The survival analysis was evaluated using the Kaplan–Meier method and compared using log-rank test. Univariate and multivariate Cox regression analyses were performed to analyze the survival data. Person correlation analysis was used to assess the correlation between REGγ (PSME3) expression and key Hippo-YAP pathway genes expression in RCC tissues derived from TGCA datasets. A value of *P* < 0.05* was considered to be statistically significant.

## Results

### REGγ is upregulated and correlated with a poor prognosis in RCC

**Fig. 1:**
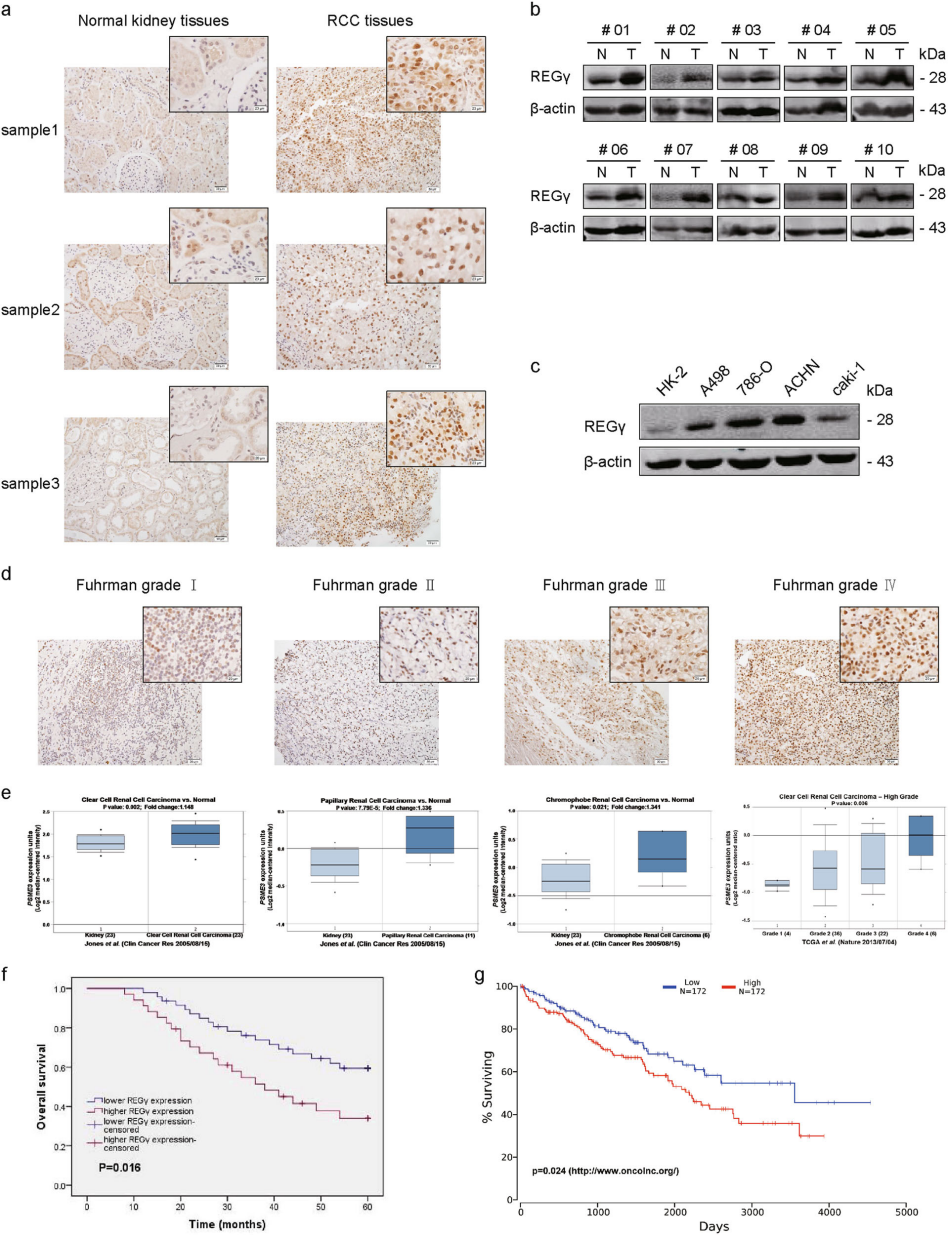
REGγ is upregulated and correlated with a poor prognosis in RCC. **a**, **b** Expression of REGγ in RCC tissues (T) and normal kidney tissues (N) as detected by IHC (**a**) and WB (**b**). **c** Expression of REGγ in four RCC cell lines (A498, 786-O, ACHN, and caki-1) and the normal renal tubular epithelial cell line (HK-2) as detected by western blot. **d** Comparison of REGγ expression in different Fuhrman grades I–IV of RCC tissue samples via IHC staining (left panel: magnification ×200, scale bar = 50 μm; right panel: magnification ×400, scale bar = 20 μm). **e** REGγ (PSME3) expression (median of expression intensity) in different pathological types and grades of RCC derived from Oncomine database (https://www.oncomine.org/). **f** Kaplan–Meier analysis of the correlation between REGγ expression and the survival time in RCC patients. Cases were classified into lower expression group and higher expression group as described in methods. **g** Prognosis of RCC (KIRC) patients with high or low expression of REGγ (PSME3) derived from OncoLnc database (https://www.oncolnc.org/)

The expression of REGγ was examined in RCC tissues and corresponding normal kidney tissues by using immunohistochemistry (IHC) and WB analysis. Results revealed that REGγ expression was significantly upregulated in RCC compared with normal kidney tissues (Fig. 1a, b). Consistently, the expression level of REGγ was also obviously higher in 4 RCC cell lines (A498, 786-O, ACHN, and caki-1) than that in the immortalized primary human proximal tubular cell line (HK-2) (Fig. 1c). In addition, we observed that REGγ staining in RCC was positively correlated with the fuhrman grade using IHC (Fig. 1d). Meanwhile, upregulation of REGγ mRNA level and the positive correlation between REGγ expression and pathological grade in RCC were also confirmed by searching the Oncomine open cancer microarray database (https://www.oncomine.org/) (Fig. 1e).

Next, we aimed to explore whether REGγ expression was associated with RCC patients’ prognosis. Statistical analyses indicated that upregulation of REGγ was significantly correlated with tumor size, T stage, Fuhrman grade, and metastasis in RCC (Table 1, *P* < 0.05). We then performed univariate and multivariate logistic regression models to analyze the correlation of REGγ expression with overall survival of RCC patients. Univariate analysis indicated that REGγ expression, tumor size, T stage, Fuhrman grade, and metastasis were associated with overall survival (Table 2, *P* < 0.05). Multivariate analysis revealed that REGγ expression, tumor size and T stage were correlated with overall survival in RCC patients (Table 2, *P* < 0.05). Kaplan–Meier analysis indicated that patients with higher expression of REGγ have a relative shorter survival time than those with lower expression of REGγ (Fig. 1f). Similarly, data from Oncolnc database (https://www.oncolnc.org/) also supported that high level of REGγ predicted a poor prognosis in RCC (Fig. 1g).

**Table 1:** Correlation between REGγ expression and clinicopathologic features in patients with RCC

Parameters	Group	*N*	REGγ expression		*P* value^a^
			Low	High	
Age (years)	<60	36	24	12	0.159
	≥60	45	23	22	
Gender	Male	43	24	19	0.668
	Female	38	23	15	
Tumor Side	Left	39	22	17	0.777
	Right	42	25	17	
Tumor Size(cm)	≤4	49	33	16	0.035*
	>4	32	14	18	
T stage	T1–2	62	41	21	0.008*
	T3–4	19	6	13	
Fuhrman	I–II	66	42	24	0.032*
	III–IV	15	5	10	
Metastasis	Negative	64	41	23	0.033*
	Positive	17	6	11	

* Statistically significant (*p* < 0.05)

^a^ *p* value from Chi-square test

**Table 2:** Univariate and multivariate cox regression analyses of REGγ expression and overall cancer survival in patients with RCC

	Univariate analysis		Multivariate analysis	
	HR (95% CI)	*P* Value	HR (95% CI)	*P* Value
REGγ expression				
Low	1.0 (Reference)		1.0 (Reference)	
High	2.126 (1.130–3.999)	0.019*	1.649 (1.327–3.287)	0.035*
Age				
<60	1.0 (Reference)		1.0 (Reference)	
≥60	1.549 (0.817–2.939)	0.180	1.232 (0.597–2.544)	0.572
Gender				
Male	1.0 (Reference)		1.0 (Reference)	
Female	1.271 (0.678–2.384)	0.455	1.461 (0.719–2.967)	0.294
Tumor side				
Left	1.0 (Reference)		1.0 (Reference)	
Right	0.93 (0.496–1.745)	0.822	0.917 (0.455–1.847)	0.807
Tumor size				
≤4 cm	1.0 (Reference)		1.0 (Reference)	
å 4 cm	2.007 (1.069–3.768)	0.030*	2.510 (1.089–5.781)	0.038*
T Stage				
T1–T2	1.0 (Reference)		1.0 (Reference)	
T3–T4	2.97 (1.489–5.921)	0.002*	3.393 (1.919–5.605)	0.045*
Fuhrman				
I–II	1.0 (Reference)		1.0 (Reference)	
III–IV	3.006 (1.475–6.374)	0.003*	1.597 (0.539–4.731)	0.198
Metastasis				
Negative	1.0 (Reference)		1.0 (Reference)	
Positive	2.349 (1.165–4.736)	0.017*	1.678 (0.898–4.033)	0.148

*CI* confidence interval, *HR* hazard ratio

* Statistically significant (*p* < 0.05)

*p* value from Cox regression analyses

Taken together, these results above suggested that REGγ served as a prognostic marker in RCC progression.

### REGγ depletion suppresses RCC cell progression in vitro

**Fig. 2:**
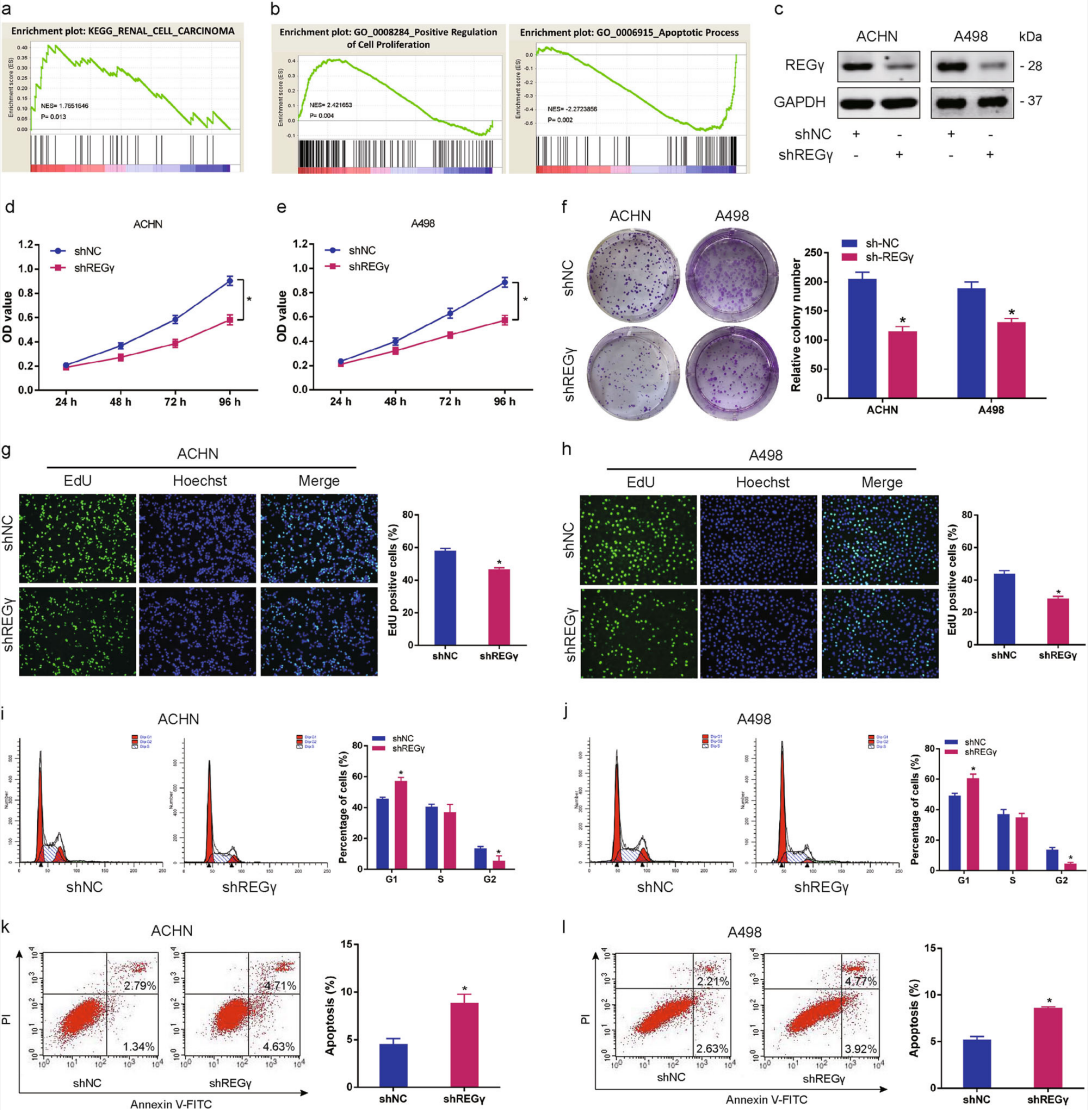
REGγ depletion suppresses RCC cells progression in vitro. **a** PSME3 upregulation is closely correlated with renal cell carcinoma revealed by KEGG pathway analysis. **b** GSEA indicated that cell proliferation and anti-apoptosis were positively correlated with elevated PSME3 expression in RCC from database GSE89563 (GO_0008284 and GO_0006915). NES, normalized enrichment score. **c** Stable knockdown of REGγ (shREGγ) in RCC cell lines (ACHN and A498) confirmed by WB. **d**, **e** Effect of shREGγ on RCC cells growth as determined by MTT assay. **f** Representative images of colony formation of RCC cells after transfection of shREGγ versus shNC. **g**, **h** Representative images of EdU incorporation assay after transfection of shREGγ versus shNC. **i**, **j** Representative flow cytometry plots of cell cycle distribution from ACHN and A498 cells transfected with shREGγ and shNC. **k**, **l** Apoptosis rate from ACHN and A498 cells after transfected with shREGγ and shNC as detected by flow cytometry. Data are shown as mean ± SD. *P < 0.05

As REGγ was upregulated in RCC tissues and cell lines, we supposed that REGγ may possess tumor-inductive properties in RCC cell biological functions. KEGG pathway analysis revealed that REGγ (PSME3) upregulation is closely correlated with renal cell carcinoma (Fig. 2a). In addition, gene set enrichment analysis (GSEA) indicated that cell proliferation and anti-apoptosis were positively correlated with elevated PSME3 expression in RCC from database GSE89563 (GO_0008284 and GO_0006915) (Fig. 2b). Next, we established RCC cell lines ACHN and A498 with stable REGγ knockdown and performed a series of functional assays (Fig. 2c). We firstly demonstrated that knockdown of REGγ effectively attenuated RCC cells growth as determined by MTT assay (Fig. 2d, e). In parallel, results of colony formation assay confirmed that the colony formation rates of RCC cells were obviously lower in REGγ silencing group than that in control group (Fig. 2f). Furthermore, data from EdU incorporation assay also indicated that the proliferation of RCC cells was significantly inhibited when REGγ was knocked down (Fig. 2g, h). Using flow cytometry to gauge the proportion of cell phase, we observed that the percentage of RCC cells was increased in G1 phase while decreased in G2 and S phase following REGγ depletion (Fig. 2i, j). We also evaluated the effect of REGγ on RCC cells apoptosis and found that knockdown of REGγ saliently facilitated RCC cells apoptosis (Fig. 2k, l).

**Fig. 3:**
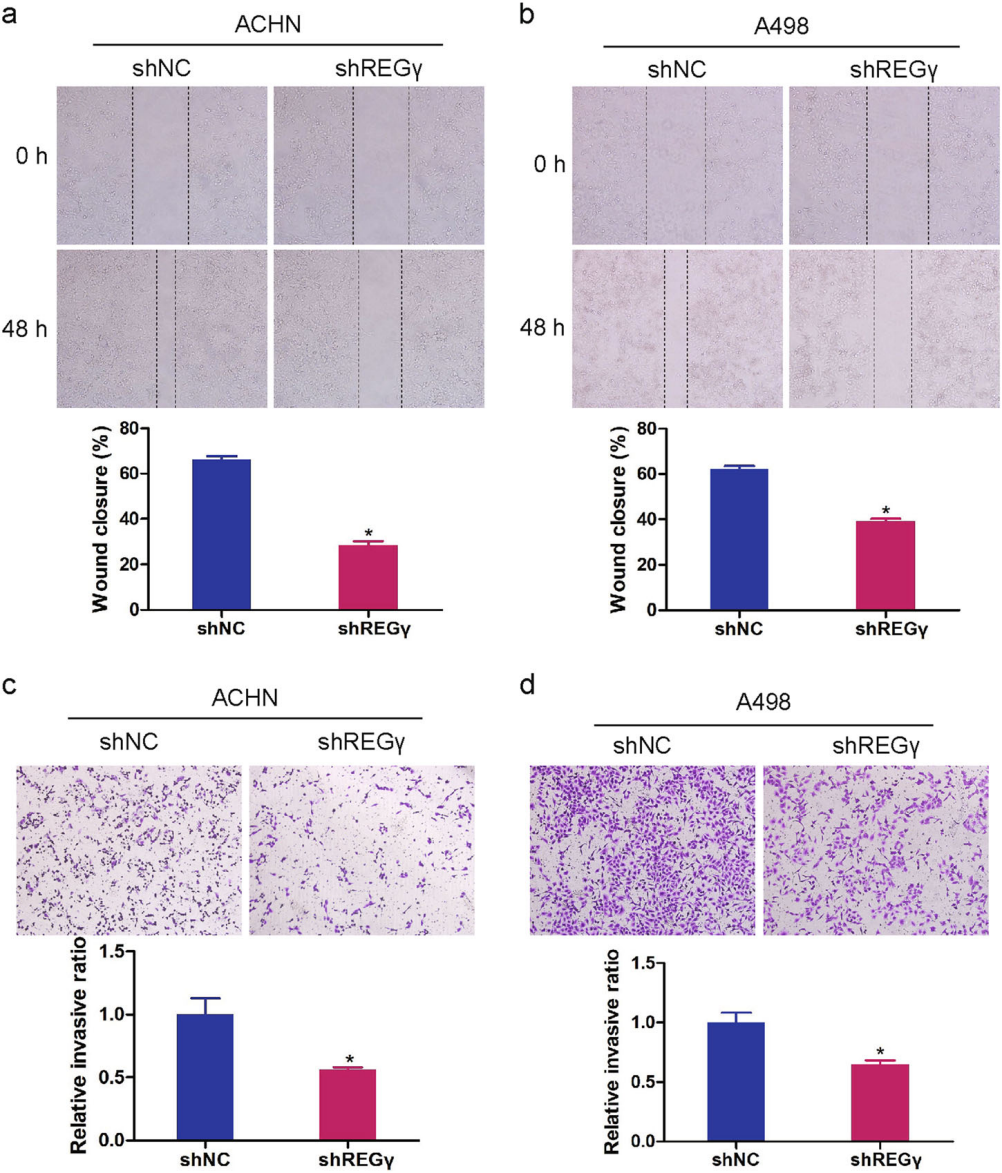
REGγ depletion inhibits RCC cells migration and invasion. **a**, **b** Representative images and the relative quantification of wound-healing assay in RCC cells transfected with shREGγ and shNC. **c**, **d** Representative images and the relative quantification of transwell invasion assay in ACHN and A498 cells transfected with shREGγ and shNC. Data are shown as mean ± SD. *P < 0.05

Moreover, we investigated the effect of REGγ on RCC cell migration and invasion. Data from wound-healing assay indicated that knockdown of REGγ significantly inhibited migration in RCC cells (Fig. 3a, b). Results of transwell invasion assay also showed that REGγ depletion markedly suppressed RCC cell invasion (Fig. 3c, d).

Taken together, these results above suggested that REGγ depletion suppressed RCC cell proliferation, migration and invasion, and enhanced cell apoptosis.

### REGγ interacts with CK1ε and promotes its degradation

**Fig. 4:**
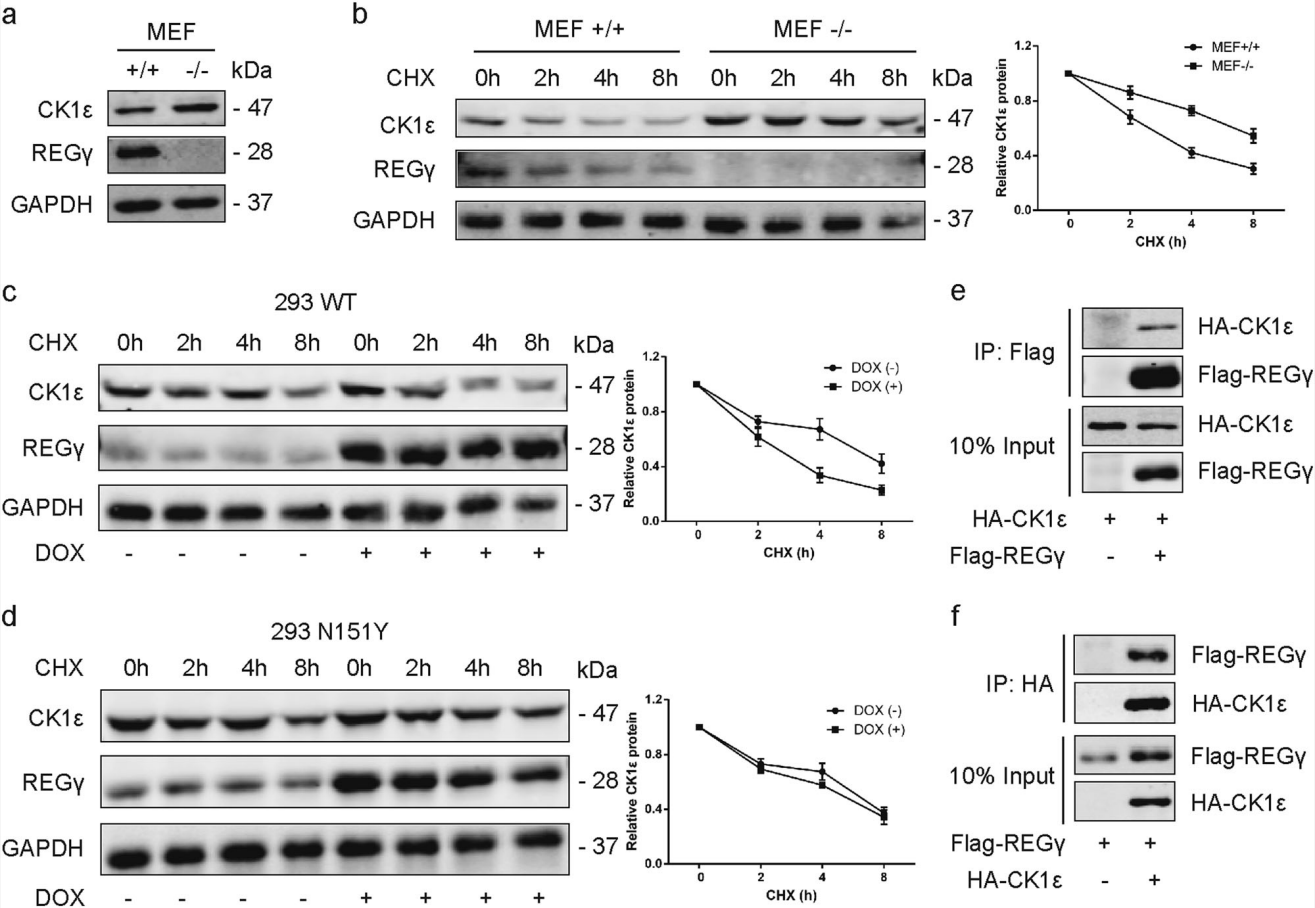
REGγ interacts with CK1ε and promotes its degradation. **a** Expression of CK1ε in REGγ+/+(REGγ wild type) and REGγ−/− (REGγ knockout) MEF cells determined by WB. **b** REGγ+/+and REGγ−/− MEFs were treated with CHX for the indicated time. The level of CK1ε was detected using WB. Relative quantification of CK1ε levels is shown. **c**, **d** CHX chase assay was performed on 293 WT cells with wild-type REGγ (**c**) and 293 N151Y cells with mutant REGγ (**d**) following DOX induced overexpression of REGγ. Relative quantification of CK1ε levels is shown. **e** Interaction between REGγ and CK1ε in 293T cells as determined by coimmunoprecipitation and WB analysis by using Flag beads following transient transfection of Flag-REGγ and HA-CK1ε. **f** Reciprocal interaction between REGγ and CK1ε as determined by coimmunoprecipitation by using HA beads as indicated. Data are shown as mean ± SD. *P < 0.05

First of all, we examined the expression levels of CK1ε in REGγ+/+(REGγ wild type) and REGγ−/− (REGγ knockout) MEF cells. The result proved that CK1ε protein level was pronouncedly elevated in REGγ−/− MEFs compared with REGγ+/+MEFs (Fig. 4a). Then, the stability of CK1ε protein in REGγ+/+and REGγ−/− MEF cells was evaluated by cycloheximide (CHX, 100 μg/ml, Amresco, Solon, OH) treatment for indicated times. Our data of WB analysis revealed that CK1ε was degraded faster in REGγ+/+MEFs than REGγ−/− MEF cells (Fig. 4b). Conversely, we applied the CHX chase assay in 293 cells inducibly expressing a wild-type (WT) REGγ or an enzymatically inactive mutant (N151Y) REGγ and found that in the presence of CHX, CK1ε decayed faster in 293 WT cells following doxycycline (DOX) induced overexpression of WT REGγ (Fig. 4c). However, the degradation of CK1ε in 293 N151Y cells did not show an obvious change following DOX treatment (Fig. 4d). Moreover, we detected the physical interactions between REGγ and CK1ε by immunoprecipitation and results revealed that the Flag-tagged REGγ successfully coimmunoprecipitated HA-tagged CK1ε (Fig. 4e). In turn, the HA-tagged CK1ε also successfully coimmunoprecipitated Flag-tagged REGγ (Fig. 4f).

These data strongly suggested that REGγ could interact directly with CK1ε and destabilize CK1ε protein with a consequent augment of its degradation.

### REGγ exerts its effect on RCC cells growth via destabilizing CK1ε

**Fig. 5:**
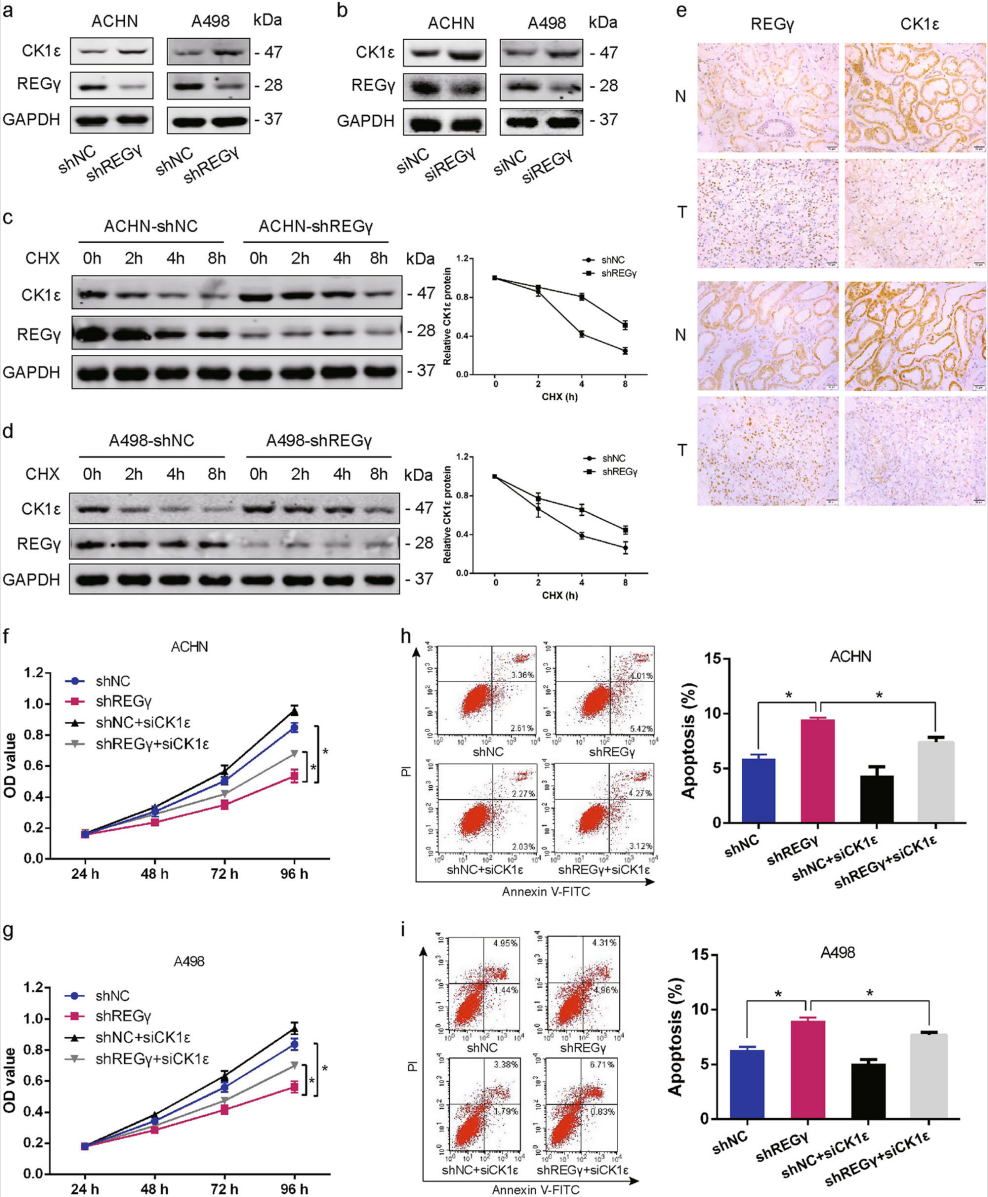
REGγ exerts its effect on RCC cells growth via destabilizing CK1ε. **a** Expression of CK1ε in RCC cells following stable knockdown of REGγ. **b** Expression of CK1ε in RCC cells following REGγ knockdown by small interfering RNA. **c**, **d** The stability of CK1ε was assessed by CHX treatment for indicated time in RCC cells following REGγ depletion. **e** The correlation between CK1ε and REGγ expression in RCC tissue samples revealed by IHC staining. Scale bar = 50 μm. **f**, **g** The suppressive effect of REGγ depletion on RCC cells growth was partly abolished by CK1ε knockdown as determined by MTT assay. **h**, **i** The promotion of REGγ depletion on RCC cells apoptosis was partly reversed by CK1ε knockdown as determined by flow cytometry. Data are shown as mean ± SD. *P < 0.05

Given that REGγ could interact with CK1ε and promote its degradation, we supposed that REGγ might modulate RCC cell progression via regulating the degradation of CK1ε in RCC cells. We observed that knockdown REGγ led to an induction of CK1ε expression in ACHN and A498 cells by WB analysis (Fig. 5a). As expected, the negative regulation of CK1ε by REGγ was also confirmed by using small interfering RNA (siRNA) to downregulate REGγ in both RCC cells (Fig. 5b). In addition, we applied CHX treatment and found that the half-life of CK1ε was markedly facilitated in RCC cells following REGγ depletion (Fig. 5c, d). Notably, the protein level of REGγ was negatively correlated with the expression of CK1ε in RCC tissue samples by IHC staining (Fig. 5e).

Taken together, these results above indicated that REGγ may destabilize CK1ε in RCC cells and the oncogenic role of REGγ in RCC maybe mediated by CK1ε. To confirm this conclusion, we transfected si-CK1ε into RCC cells to abolish the upregulation of CK1ε following REGγ depletion. Subsequently, cell proliferation and apoptosis were determined by MTT assay and flow cytometry, respectively. MTT assay revealed that knockdown of CK1ε partly rescued the suppressive effect of REGγ depletion on RCC cells proliferation (Fig. 5f, g). In parallel, data from flow cytometry certified that the effect of REGγ depletion on RCC cells apoptosis was also blocked by CK1ε knockdown (Fig. 5h, i). In brief, these results collectively suggested that REGγ exerted its oncogenic role via destabilizing CK1ε in RCC.

### REGγ/CK1ε axis modulates Hippo signaling in RCC

**Fig. 6:**
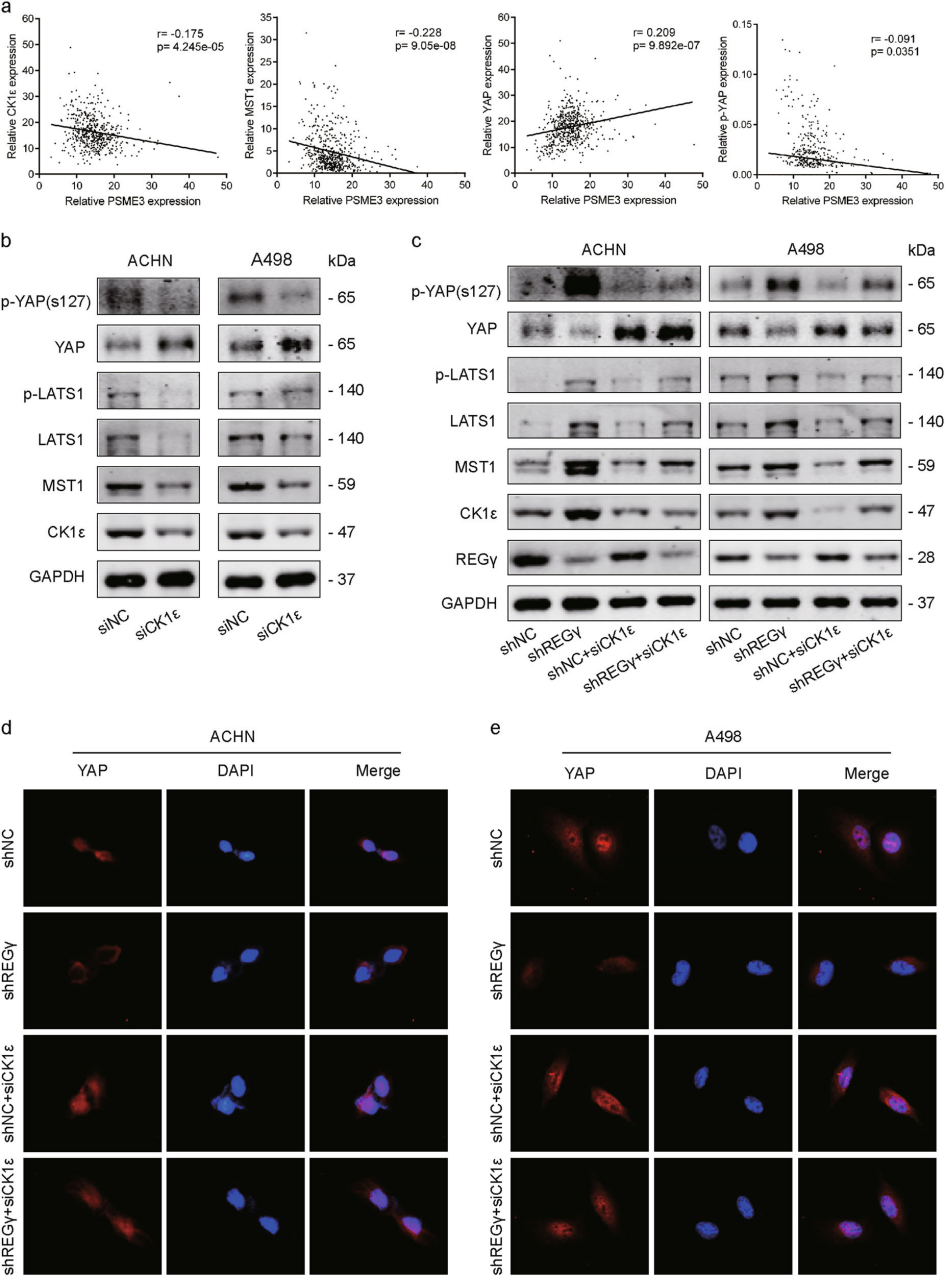
REGγ/CK1ε axis modulates Hippo signaling in RCC. **a** The correlations between REGγ (PSME3) expression and key Hippo pathway genes from TCGA datasets. **b** Effect of CK1ε knockdown on Hippo signaling in RCC cells. **c** Effect of REGγ depletion on Hippo signaling in RCC cells and that effect was abolished by CK1ε knockdown. **d**, **e** The expression level and localization of YAP in RCC cells following REGγ depletion or/and CK1ε knockdown by IF staining

Previous report demonstrated that MST1 was one of the substrates of CK1ε and that CK1ε might mediate Hippo signaling pathway in cancer progression and development^15^. We hypothesized that the REGγ/CK1ε axis might exert its function by partly modulating Hippo signaling in RCC. Firstly, the correlations between REGγ (PSME3) expression and key Hippo pathway genes from TCGA datasets were analyzed. Results showed that PSME3 expression is negatively correlated with CK1ε, MST1, and p-YAP, while positively correlated with YAP in RCC tissues (Fig. 6a). Then, the protein levels of several important members from Hippo signaling were determined by WB analysis following CK1ε knockdown in ACHN and A498 cells. The result showed that p-YAP, p-LATS1, LATS1, and MST1 were significantly suppressed by CK1ε knockdown in RCC cells (Fig. 6b). Furthermore, the effect of REGγ on Hippo signaling in RCC was also evaluated. Our results indicated that REGγ depletion activated Hippo signaling pathway in RCC cells while the activation by REGγ depletion was effectively abolished following CK1ε knockdown in both RCC cells via WB analysis (Fig. 6c). It is well established that the Hippo signaling played its vital role through mainly regulating the phosphorylation and localization of its target protein YAP in cancer development^26^. By using immunofluorescence analysis, we observed that REGγ depletion obviously decreased the nuclear accumulation of YAP in RCC cells. However, knockdown of CK1ε effectively reversed the effect of REGγ on the localization of YAP in RCC cells (Fig. 6d, e).

Taken together, these results revealed that REGγ/CK1ε axis might exert its effect via modulating Hippo signaling in RCC.

### REGγ depletion suppresses RCC cell tumorigenesis in vivo

**Fig. 7:**
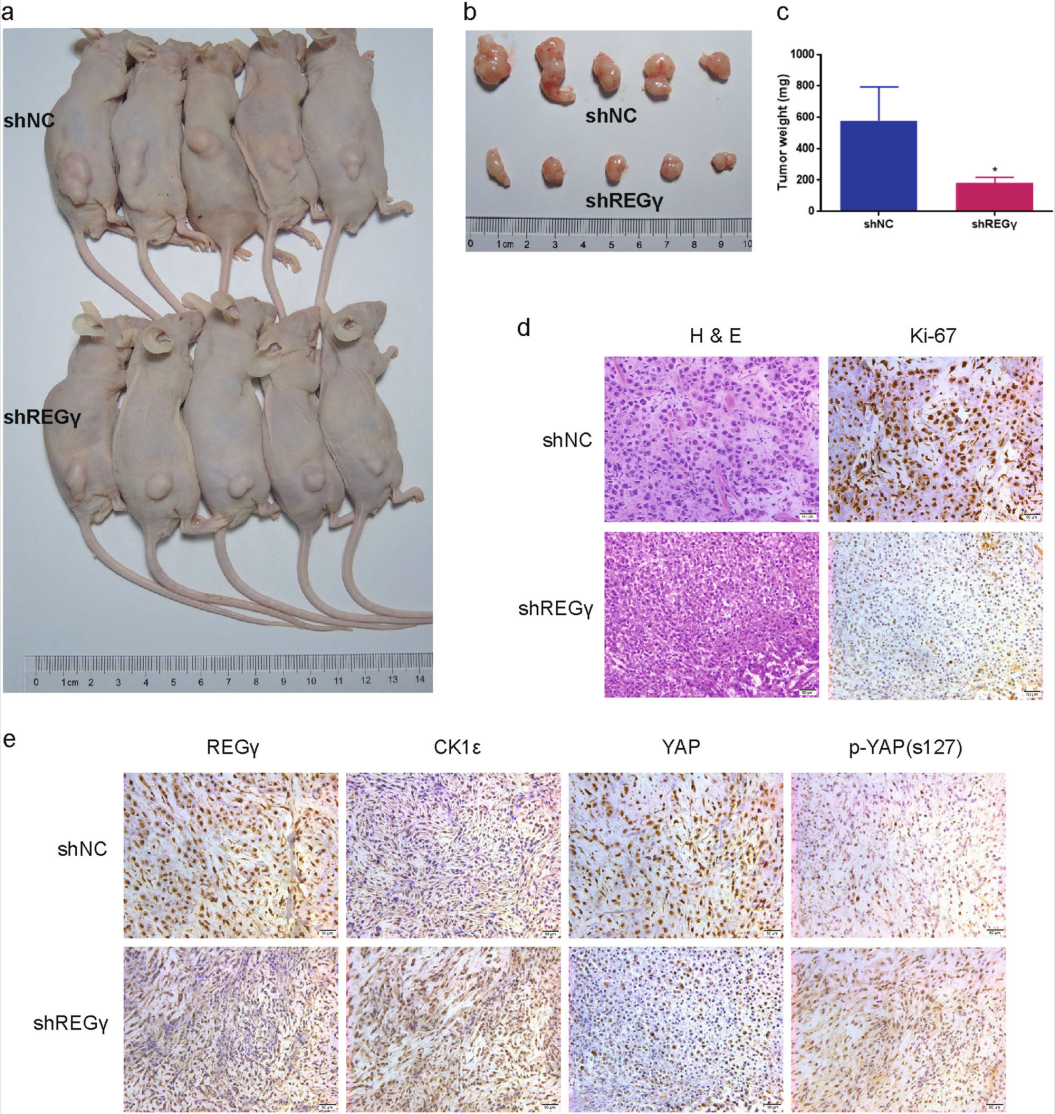
REGγ depletion suppresses RCC cells growth in vivo. **a** ACHN shNC and shREGγ cells were injected into the flanks of BALB/c nude mice. Representative photograph of nude mice bearing the xenograft tumors was shown following four weeks. **b**, **c** Representative images (**b**) and average weight (**c**) of the isolated xenograft tumors from shNC or shREGγ group were shown. **d** Representative images of H&E staining and Ki-67 immunohistochemical (IHC) detection of the excised tumors derived from nude mice. **e** IHC staining of REGγ, CK1ε, YAP, and p-YAP in the excised tumors derived from nude mice. Scale bar = 50 μm

To further validate the effect of REGγ on RCC cells tumorigenesis in vivo, we established the xenograft animal model by subcutaneously injecting ACHN shNC and shREGγ cells into the flanks of BALB/c nude mice. The xenograft tumor growth was estimated following four weeks of tumor implantation. As expected, REGγ depletion saliently attenuated the growth of tumors in nude mice compared with control group (Fig. 7a, b). Similarly, the average weight of xenograft tumors in shREGγ group was also markedly lower than that in shNC group (Fig. 7c). In addition, the significant lower proportion of Ki-67 positive cells in xenograft tumors derived from shREGγ group compared to the shNC group were observed by IHC analysis (Fig. 7d). Furthermore, the expression levels of REGγ, CK1ε, YAP, and p-YAP in xenograft tumors were analyzed by IHC and the result indicated that CK1ε and p-YAP were upregulated while YAP was downregulated in xenograft tumors of shREGγ group compared with shNC group (Fig. 7e).

**Fig. 8:**
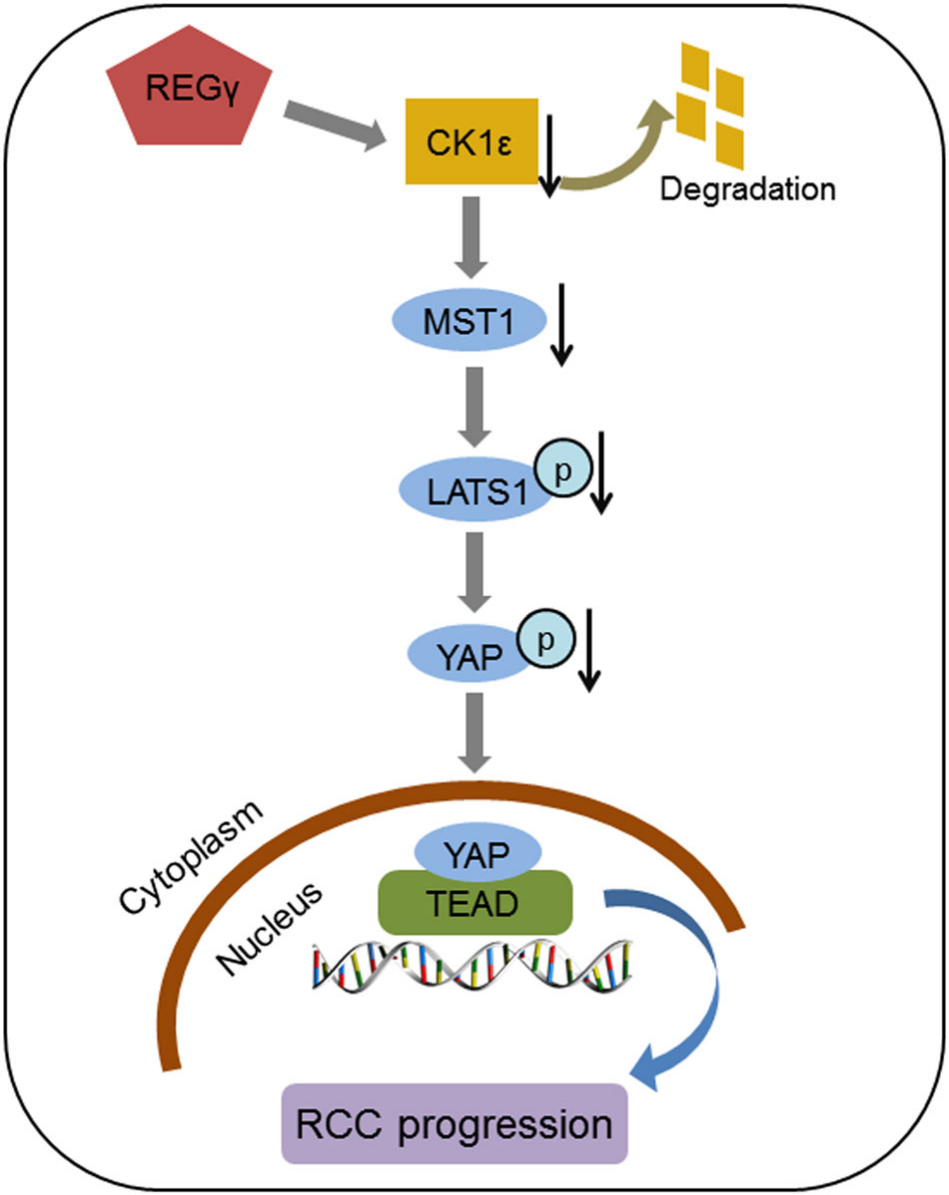
Schematic model for the effect of REGγ/CK1ε axis on RCC progression. Upregulation of REGγ in RCC promotes the degradation of CK1ε to downregulate the level of MST1. The MST1 downregulation decreases the phosphorylation level of LATS1 and YAP, which leads to the nucleus accumulation of YAP in RCC cells and finally promote RCC tumor progression

In conclusion, these results confirmed that REGγ depletion suppressed RCC cell progression via CK1ε mediated modulation of Hippo signaling in vivo. The effect of REGγ/CK1ε axis on RCC progression was summarized by a schematic model (Fig. 8).

## Discussion

In recent years, the role of REGγ in tumorigenesis has gained massive attention and abundant publications have demonstrated REGγ as an oncogene in various human cancers namely prostate cancer^27^, pancreatic cancer^28^, and colon cancer^29^. In the present study, we found for the first time that the expression of REGγ was dramatically upregulated in RCC tissue samples and cell lines compared with normal controls. Statistical analysis showed that upregulation of REGγ was significantly correlated with tumor size, T stage, Fuhrman grade, and metastasis in RCC patients. Meanwhile, high expression of REGγ was correlated with a poor prognosis in patients with RCC revealed by Kaplan–Meier analysis. Then, data from functional assays indicated that silencing of REGγ sufficiently attenuated RCC cells proliferation, colony formation, and cell cycle transition accompanied by the apoptosis induction. In addition, we also observed that REGγ depletion significantly suppressed RCC cells migration and invasion. These results suggest that REGγ functions as an oncogenic protein in the development of RCC.

As a member of the 11S proteasome activator family, REGγ was originally recognized to just promote the degradation of unfolded proteins and model peptide substrates by activating the 20S proteasome^6, 30^. However, this view was challenged when Li et al. reported that REGγ could degrade the intact protein SRC-3^7^. Following this report, more proteins such as p21, smurf1, and c-Myc were demonstrated to be the substrates of REGγ^20, 31, 32^. Among that, CK1δ, which is 98% similar with CK1ε in structure, was also identified as a substrate of REGγ^21^. In our study, we observed that CK1ε was induced in REGγ knockout (REGγ−/−) MEFs compared with normal (REGγ+/+) MEFs and CK1ε was degraded faster in REGγ+/+MEFs following CHX treatment. Then, we proved that REGγ could physically interact with CK1ε. The stability of CK1ε regulated by REGγ was also validated in RCC cell lines ACHN and A498. Specifically, data from IHC analysis attested that the expression of REGγ and CK1ε was negatively correlated in RCC tissues. Furthermore, the functional effect of REGγ on RCC cells proliferation and apoptosis was proved to be mediated by CK1ε through rescue assays. These results above revealed that REGγ may be involved in the development of RCC by interacting with and enhancing the degradation of CK1ε.

The Hippo signaling pathway, initially identified in *Drosophila*, is a conserved regulator of tissue growth and organ size^33^. Recently, it is defined that the Hippo signaling was very imperative to various biological functions, such as cell proliferation, apoptosis, differentiation, and development^34^. Mechanistically, the Hippo pathway in mammals is a kinase cascade in which MST1/2 phosphorylates and activates LATS1/2, which then phosphorylates YAP/TAZ to promote its cytoplasmic retain. The phosphorylation and localization of YAP/TAZ will regulate the expression of a wide range of genes that are involved in cell proliferation, survival, and migration^35^. Previous publications demonstrated that the expression of LATS1 was markedly reduced while YAP was overexpressed in RCC, indicating the pivotal role of Hippo signaling in the development of RCC^36, 37^. In this study, we reported that depletion of REGγ pronouncedly activated Hippo signaling pathway in RCC cells and the effect was effectively abolished following knockdown of CK1ε in a rescue assay. Meanwhile, REGγ depletion decreased the nuclear accumulation of YAP in RCC cells and knockdown of CK1ε reversed this effect. These results confirmed that REGγ may modulate Hippo signaling pathway in a CK1ε dependent manner in RCC. The regulation of Hippo signaling pathway by REGγ/CK1ε axis in RCC was also verified in vivo by IHC analysis on tumor tissues from the xenograft animal model.

In *Drosophila*, the CK1δ/ε homolog *discs overgrown (dco)* was reported as a tumor suppressor and positioned in the Hippo pathway upstream of *dachs* by its regulation of the Hippo pathway downstream target genes^38^. In our study, we found that CK1ε may regulate Hippo signaling via MST1/LAST1/YAP axis and the expression of YAP was negatively regulated by CK1ε in RCC. Interestingly, Zhao et al. reported that CK1ε can activate the phosphodegron and permit β-TRCP binding to promote YAP degradation^39^. Thus outcomes suggested that CK1ε may regulate Hippo signaling via some other manners, which requires further investigation in RCC. At present, target therapy is the first-line treatment approach for metastatic RCC (mRCC). The molecular targets used in clinic include vascular endothelial growth factor (VEGF), mammalian target of rapamycin (mTOR), platelet-derived growth factor (PDGF), and so on^40^. However, the outcome is still poor. It is necessary to identify novel targets for mRCC treatment. Our study showed that REGγ was involved in RCC development and correlated with the prognosis of RCC patients. These results suggest that REGγ may serve as a novel molecular marker for the diagnosis and prognosis prediction and may act as a therapeutic target in RCC patients.

In summary, we firstly reported that REGγ was upregulated and correlated with a poor prognosis in RCC. Depletion of REGγ abrogated RCC cells growth in vitro and in vivo. REGγ could physically interact with CK1ε and promote its degradation. Knockdown of REGγ activated the Hippo signaling pathway via stabilizing CK1ε in RCC. Taken together, our findings presented a novel insight to the oncogenic role of REGγ in the development of RCC and thus may help us to identify novel approaches for RCC treatments.
